# Clustering of fluoride and phosphate ions in bioactive glass from computer simulation

**DOI:** 10.1098/rsta.2022.0345

**Published:** 2023-10-16

**Authors:** Jamieson K. Christie

**Affiliations:** Department of Materials, Loughborough University, Loughborough LE11 3TU, UK

**Keywords:** bioactive glass, clustering, molecular dynamics, dissolution

## Abstract

In order to understand the nature of ionic clustering in bioactive glass compositions, computer simulation was used to model four different compositions of bioactive glass with various amounts of flouride and phosphate. Fluoride ions were chemically bonded only to sodium and calcium, creating regions rich in fluoride and modifiers, and fluoride clustering was seen to be present in all compositions. The majority of phosphate groups are present as orthophosphate and phosphate clustering is also seen, and shown to be stronger in compositions with a lower phosphate content.

This article is part of the theme issue ‘Exploring the length scales, timescales and chemistry of challenging materials (Part 1)’.

## 1. Introduction

Bioactive glass undergoes a series of chemical reactions when implanted into the body, which includes the release of ions during its degradation, and the formation of a surface layer of apatite. Bioglass 45S5 was the first composition to be developed [1] and has been used clinically. The glass structure is amorphous and so not restricted to specific stoichiometries, and it is also able to incorporate a wide range of ions [2], implying that bioactive glass could be used as a material for controlled release of therapeutically relevant ions, in concert with its expected functions of promoting bone repair and regrowth.

The composition of any glass affects its atomic structure and the two together affect the behaviour of the glass in the body. To optimize bioactive glass for specific applications, therefore, a full understanding of the features which affect its bioactivity is necessary. The network connectivity (NC), i.e. the mean number of bridging oxygen atoms per network former, is well known to be a good predictor of bioactivity [3,4]. Glasses with lower NC tend to be more able to react with the physiological environment, hence are more bioactive.

There are other structural features that are also potentially relevant to bioactvity. One possibility is that the ions in the glass might not be uniformly arranged throughout the material, that is, they might aggregate or cluster [5–9]. The effect this might have on the bioactivity of the glass is not completely clear, but it is likely to lead to regions of the glass that have locally higher or lower NC than the average, that is, the formation of heterogeneities [10]. It is possible that these regions are related to the rigid and floppy regions of the glass described by Phillips & Thorpe [11,12].

In this paper, we use computer simulation to study different compositions of bioactive glass with more or less phosphate and fluoride content. These two ions are particularly relevant. Fluoridated bioactive glass has potential applications in dentistry and the local bonding of fluoride ions has been linked to clustering in glass compositions containing it [10,13]. Phosphate tetrahedra are usually present as unconnected orthophosphate tetrahedra [14] but the full extent of phosphate clustering is not always clear. We characterize the causes of the clustering in terms of the local structure around fluoride and phosphate, in order to illuminate further study of clustering in these glasses.

## 2. Methodology

Four different compositions were studied, and they and their densities are listed in table 1.

**Table 1.** The compositions and densities of the glasses simulated here.

	(mol %)					
name	${\mathrm{SiO}}_{2}$	${\mathrm{Na}}_{2}O$	CaO	$P_{2}O_{5}$	${\mathrm{CaF}}_{2}$	density ($g\;{\mathrm{cm}}^{-3}$)
LFLP	46.20	19.30	26.90	2.60	5.00	2.737 [13]
LFHP	36.41	28.28	24.74	6.04	4.53	2.749 [15]
HFLP	46.20	24.30	6.90	2.60	20.00	2.599 [13]
HFHP	31.37	24.36	21.31	5.21	17.76	2.79 [15]

These compositions were chosen because they are related to 45S5, and experimental densities were available. They have low or high (L/H) fluoride (F) and low or high (L/H) phosphate (P) content, and are hence labelled with L or H before F and P. The network connectivities are not all the same, but they are likely all in the bioactive region. The LFHP and HFHP compositions are part of a series chosen to have the same NC [15], but this is calculated assuming that all phosphate is present as orthophosphate [16]. This might be true experimentally, but we will see later that it is not true for our simulated models. For each composition, five independent simulations were run, and unless otherwise stated, the results are averaged over all five.

For each model, about 3000 atoms of the appropriate composition and experimental density (table 1) were placed randomly into a periodic cubic box, subject to the constraint that no two atoms were placed closer than approximately 80--90% of their typical bond lengths. After a 50 ps run in the NVT ensemble at 3000 K during which the motion of the atoms was constrained to be less than 0.025 A in each timestep, the simulation was then run in a series of NVT ensembles for 150 ps each without constraints on atomic motion, starting at a temperature of 3000 K, and then decreasing by 150 K for each subsequent run. This corresponds to a cooling rate of $1\;K\;{\mathrm{ps}}^{-1}$. After the 150 ps run at 300 K, a subsequent NVT production run of 1 ns at 300 K was performed, and all results are averaged over this production run.

The LAMMPS [17] classical molecular dynamics code was used for all simulations. The timestep was 1 fs, and the particle–particle particle-mesh method was used to evaluate the long-range electrostatic forces. The interatomic potentials are the partial-charge non-polarizable Teter potentials [13], consisting of a Buckingham term for the short-range interactions, and a Coulomb term for the electrostatics. As no short-range term existed for P-F interactions, this was set equal to the P-O term, as a first approximation.

## 3. Results and discussion

**Figure 1.**
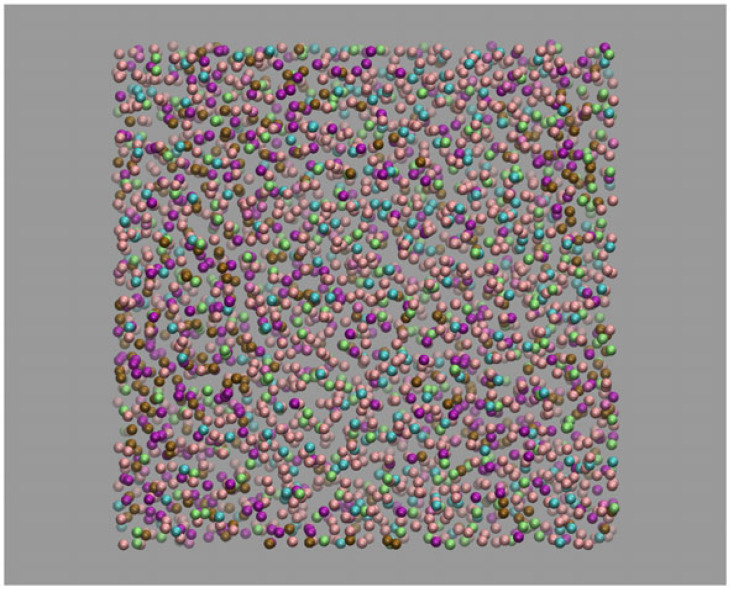
A snapshot of one model of the HFHP composition as a typical example. Oxygen atoms are pink, phosphorus atoms are orange, silicon atoms are cyan, sodium atoms are purple, calcium atoms are green and flourine atoms are brown. (Online version in colour.)

Although the clustering of ions occurs at medium-range length scales, it is rooted in the local bonding of each species, which we characterize here. A snapshot of one model of the HFHP composition as a typical example is shown in figure 1.

### (a) Flouride environment

**Figure 2.**
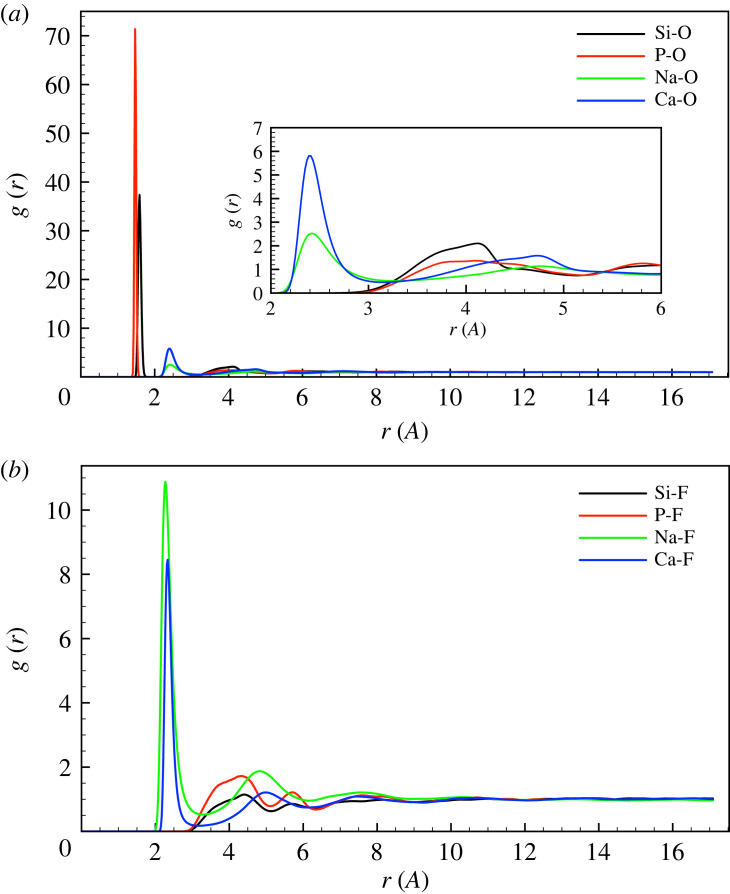
The partial pair-distribution functions g(r) for (*a*) cation-oxygen and (*b*) cation-flouride pairs in the HFHP composition as a typical example. (Online version in colour.)

The partial pair-distribution functions g(r) are shown for cation-oxygen pairs in figure 2*a* and for cation-flourine pairs in figure 2*b*, for the HFHP composition as a typical example. For all compositions, the bond lengths are given in table 2 and coordination numbers are given in table 3.

**Table 2.** The bond lengths of the glasses simulated here. They are calculated as the maximum of the g(r) distribution described by a histogram of bin width 0.02 Å; hence the errors are ±0.01 Å.

	bond length (Å)			
bond	LFLP	LFHP	HFLP	HFHP
Si-O	1.59	1.59	1.59	1.59
P-O	1.47	1.47	1.47	1.47
Na-O	2.41	2.41	2.43	2.43
Ca-O	2.39	2.39	2.39	2.39
Na-F	2.25	2.25	2.29	2.27
Ca-F	2.33	2.33	2.33	2.33

**Table 3.** The coordination numbers of the glasses simulated here. They are calculated as the integral of the g(r) distribution up to a cut-off of 2.0 Å for Si-O, Si-F, P-O and P-F and a cut-off of 3.2 Å for Na-O, Na-F, Ca-O and Ca-F.

	coordination numbers			
bond	LFLP	LFHP	HFLP	HFHP
Si-O	4.00	4.00	4.00	4.00
P-O	4.00	4.00	4.00	4.00
Na-O	5.39	5.57	2.88	3.70
Ca-O	6.12	6.29	4.96	5.51
Na-F	0.83	0.64	3.09	3.70
Ca-F	0.48	0.33	1.70	5.51
Na-(O,F)	6.23	6.21	5.98	6.31
Ca-(O,F)	6.60	6.62	6.66	6.90
% Na-O	86.6	89.7	48.2	58.7
% Ca-O	92.8	95.1	74.5	79.8

There are sharp Si-O and P-O peaks at approximately 1.6 Å and 1.5 Å, respectively (figure 2*a*), corresponding to the chemical bonds between those atoms. The Si-O and P-O coordination numbers are 4.0 (table 3), showing that the first coordination shell around both network formers is composed exclusively of oxygen ions. The lack of Si-F or P-F bonds at the same distances show that these are absent, or possibly occur at very low amounts, as seen experimentally [10,18,19] and in simulation [9,13,20] for related glasses.

By contrast, the modifiers show peaks in the pair-distribution function to both oxygen and fluoride (figure 2*b*) and the first coordination shell comprises oxygen and fluoride in mixed amounts (table 3). The coordination of sodium and calcium is between six and seven for all compositions, with first coordination shells comprising both oxygen and fluoride ions (table 3). Higher-fluoride compositions have more fluoride in the first coordination shell, and sodium seems to bond to more fluoride than calcium (table 3).

### (b) Phosphate environment

Silicon and phosphorus are both at the centre of tetrahedra with four neighbouring oxygen atoms, one at each corner of the tetrahedron. At larger length scales, however, the environment of the ${\text{PO}}_{4}$ tetrahedra is different from that of the ${\text{SiO}}_{4}$ tetrahedra. While the silicate tetrahedra form a connected three-dimensional network, the phosphate tetrahedra exist mostly as isolated orthophosphate $Q^{0}$ ${\text{PO}}_{4}$ tetrahedra. The exact amount of such tetrahedra has been the subject of discussion, as $Q^{1}$ ${\text{PO}}_{4}$ tetrahedra, i.e. phosphate tetrahedra with one bridging oxygen atom connecting them to the rest of the glass network, are seen across very many simulations [21,22] but only some experiments [23]. They appear to be more common for compositions with higher silicate and phosphate content than studied here [24,25].

In this work, we have computed the $Q^{n}$ distribution of the phosphate groups and these are shown in table 4; while most of the phosphate groups are orthophosphate, there is a substantial proportion of $Q^{1}$ and more connected phosphate groups. This is very likely an overestimate compared with experimental values for two reasons. Firstly, rigid-ion interatomic potentials such as those used here lead to more connected phosphate groups than polarizable potentials [21], but unfortunately no set of polarizable potentials exists for this system. Secondly, the phosphate $Q^{n}$ distribution is more sensitive to the cooling rate than the silicate $Q^{n}$ distribution [26]; specifically, the number of $Q^{0}$ increases with slower cooling rate. Although the cooling rate is fairly good here for computer simulation, it is of course several orders of magnitude faster than those used experimentally, and more realistic cooling rates could, if obtainable in simulation, reduce this overestimate.

**Table 4.** The $Q^{n}$ distributions and network connectivities of the phosphorus atoms for the glasses simulated here.

	%				
	$Q^{0}$	$Q^{1}$	$Q^{2}$	$Q^{3}$	NC(P)
LFLP	56.8	37.5	5.7	0.0	0.489
LFHP	69.5	28.1	2.4	0.0	0.329
HFLP	45.3	38.7	13.2	2.7	0.732
HFHP	70.8	25.8	3.3	0.2	0.329

**Figure 3.**
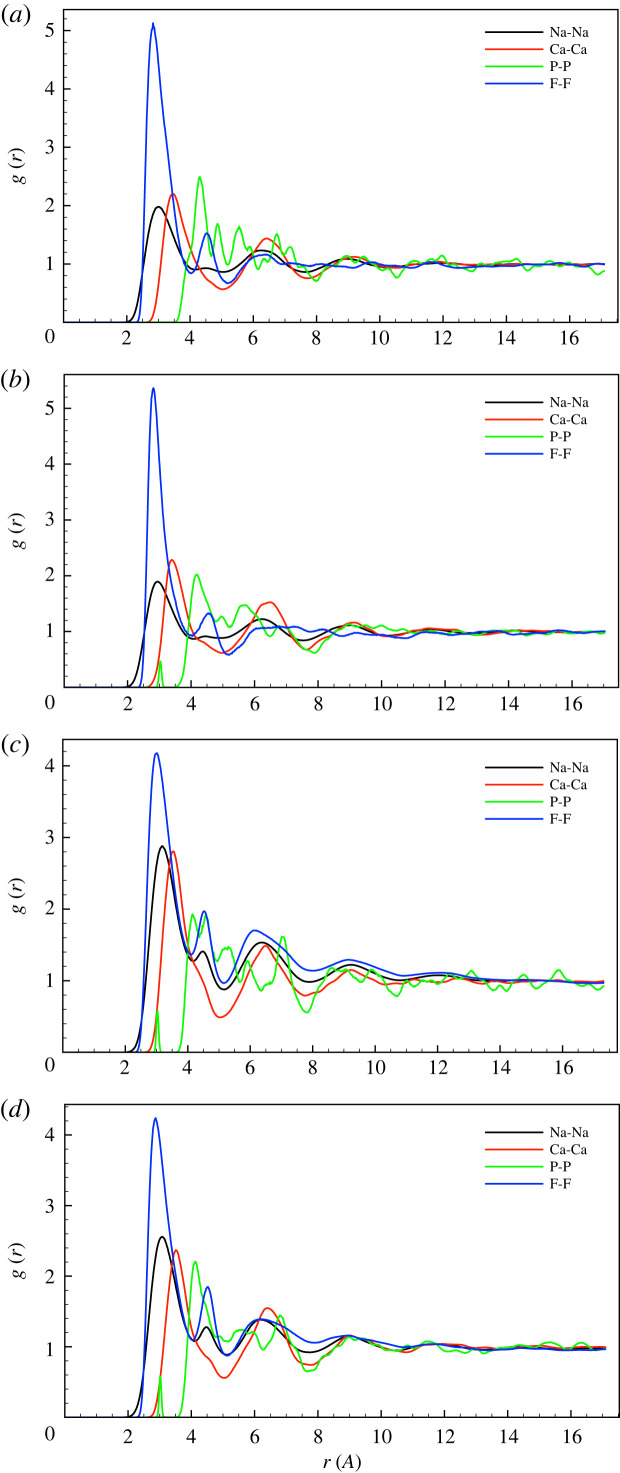
The partial pair-distribution functions $g_{\alpha \alpha }(r)$ for α=Na, Ca, P, F for the (*a*) LFLP, (*b*) LFHP, (*c*) HFLP and (*d*) HFHP compositions. (Online version in colour.)

Almost all of the connected phosphate tetrahedra are connected through P-O-Si bonds, and the number of P-O-P bonds is very small (as a proportion of P-O-X bonds, where X is any other species, these are 1.5% for HFHP, 1.6% for LFHP, 0.8% for HFLP and 0 for LFLP), and as expected a little larger in the high-phosphate compositions. These bonds can be seen in the small peak at about 3.0 Å in the P-P partial pair-correlation functions in figure 3*b*–*d*, implying a very small amount of ${\text{P}}_{2}{\text{O}}_{7}$ groups.

The extent to which phosphate groups cluster together in these and related glass compositions is not yet completely understood. The results obtained here show phosphate clustering at relatively low phosphate content, in contrast to previous simulations in which phosphate clustering was visible only at compositions containing 12 mol% phosphate [27]. Other work has suggested the presence of phosphate clustering at low phosphate content [14], even for phosphate contents as low (2.6 mol %) as the amounts seen in 45S5 and our LFLP and HFLP compositions [23]. More recent work has directly imaged phosphate clustering at compositions at higher phosphate content than 45S5 but similar to that in our LFHP and HFHP compositions [28,29]. It is clear that there is no general value of phosphate content for the onset of phosphate clustering.

For compositions of glass that have higher silicate content than considered here (hence compositions which are not bioactive), it seems possible that both increased phosphate clustering, and a separation of sodium (which clusters with silicon) and calcium (which clusters with phosphorus), exist [10].

### (c) Ionic clustering

The Na-Na, Ca-Ca, P-P and F-F partial pair-correlation functions are shown in figure 3, and the clustering ratio r for these pairs of ions is given in table 5.

**Table 5.** The clustering ratio r of various ions for the glasses simulated here.

	clustering ratio r			
	LFLP	LFHP	HFLP	HFHP
Na-Na	1.21	1.11	1.56	1.41
Ca-Ca	1.14	1.14	1.36	1.13
P-P	2.29	1.46	2.40	1.59
F-F	2.25	2.20	1.96	1.81

The clustering ratio r is defined as [22,30]

3.1 $$r_{X-Y}=\frac{N_{X-Y,\mathrm{MD}}}{N_{X-Y,\mathrm{hom}}}=\frac{CN_{X-Y}+\delta_{X-Y}}{\left(4/3)\pi r_{c}^{3}(N_{X}/V_{\text{box}}\right)},$$

where $CN_{X-Y}$ is the observed X−Y coordination number, $\delta_{X-Y}$ is 1 if X=Y and 0 otherwise, $r_{c}$ is the distance cut-off at which the X−Y coordination numbers are calculated, set to 5.0 Å in this work, $N_{X}$ is the number of atoms of species X in the simulation box, which has a volume $V_{\text{box}}$. As r is a measure of the ratio of the observed coordination number to that expected if the atoms were distributed homogeneously, values of r greater than 1 indicate preferential clustering of atoms.

It is clear from table 5 than both phosphorus and fluoride cluster in these glasses, and that the clustering ratio for a particular species is higher in the compositions for which the content of that species is lower, as was also observed for other bioactive and related glass compositions [6,31].

By contrast, sodium and calcium have a much smaller amount of clustering, which is usually (but not always) higher in the high-fluoride compositions, presumably because the increased amount of fluoride creates regions rich in fluoride and modifier ions, which reduces the Na-Na and Ca-Ca distances, so increasing the clustering ratio. The fluoride clustering is clear from the strong first F-F peak at r∼3.0 Å in the partial pair-correlation functions (figure 3), larger than the Na-Na or Ca-Ca peaks.

## 4. Conclusion

Four different compositions of bioactive glass with various amounts of flouride and phosphate were computationally modelled and the ionic clustering examined. No Si-F or P-F bonds were found, and fluoride was exclusively bonded to sodium and calcium, the network-modifying cations present in these compositions. This creates regions of the glass rich in fluoride and modifiers, and this is likely to be the reason for the presence of fluoride clustering in all compositions. The majority of phosphate groups are present as $Q^{0}$ orthophosphate and, although the amount of $Q^{1}$ and other phosphate groups is likely overestimated by our simulations, phosphate clustering is also observed, and shown to be stronger at compositions with low phosphate content, in agreement with some experimental observations.

## Data Availability

This article has no additional data.
